# Low-grade Leiomyosarcoma of the Cavernous Sinus in an HIV Positive Patient: Case Report

**DOI:** 10.7759/cureus.6758

**Published:** 2020-01-23

**Authors:** Laura Morales R, Angelina Álvarez, Maria Paulina Noreña, Felipe Torres, José Esguerra

**Affiliations:** 1 Radiation Oncology, Instituto Nacional de Cancerologia, Bogota, COL; 2 Radiation Oncology, Instituto Nacional de Cancerología, Bogota, COL; 3 Radiation Oncology, Centro de Control de Cancer, Bogota, COL

**Keywords:** leiomyosarcoma, primary intracranial leiomyosarcomas, h.i.v, cancer radiotherapy

## Abstract

Primary leiomyosarcomas of the central nervous system are extremely rare tumors, with few cases reported in the literature. In this article we report the case of a patient with an intracranial leiomyosarcoma of the cavernous sinus. This is the case of a 23-year-old man with a history of human immunodeficiency virus (HIV) and Epstein-Barr virus infection, with clinical picture of headache and left palpebral ptosis, who underwent nuclear magnetic resonance imaging that showed a lesion that occupied the cavernous sinus. Excisional biopsy reported fusocellular mesenchymal neoplasm with smooth muscle differentiation by immunohistochemistry compatible with low-grade leiomyosarcoma. The patient was initially taken to a partial resection, without treatment. Subsequently, the patient presented progression of his disease, so the area of neurosurgery considered that the lesion was unresectable due to its location and the risk of sequelae. It was then decided to treat the patient with intensity-modulated radiation therapy technique external radiotherapy. At six months of treatment, the patient continues asymptomatic with a stable disease.

## Introduction

Leiomyosarcomas are rare tumors derived from soft tissues, smooth muscle cells. They represent between 1% and 4% of soft tissue sarcomas of the head and neck. These tumors can originate from structures such as vascular walls [1], even so, its presentation at the level of the central nervous system is quite uncommon since reports such as Paulus et al. found that less than <1% of brain biopsies are compatible with leiomyosarcoma (three out of 25,000 brain tumors) [2]. In addition to this, most cases with intracranial involvement correspond to metastatic disease of other primary sites such as the gastrointestinal tract, uterus or subcutaneous cellular tissue, making its presentation as an intracranial primary tumor exceptional with less than 50 cases reported [3]. In recent years, there has been an increase in its incidence, probably associated with human immunodeficiency virus (HIV) [4].

## Case presentation

This article reports the case of a 23-year-old male patient diagnosed with HIV, who started two years ago with a clinical picture of hemicranial headache and left palpebral ptosis. He underwent a nuclear magnetic resonance imaging (MRI), which showed a solid lesion that occupied the cavernous sinus on the left side. He was taken to an excisional biopsy that reported fusocellular mesenchymal neoplasm with smooth muscle differentiation. With this result, he underwent a partial resection, according to the surgical description, and he was not given any adjuvant treatment, leaving the patient under follow-up. A year later, a follow-up contrasted cerebral MRI showed a receiving lesion of the contrast medium with an intimate relationship to the cavernous sinus with extra axial extension towards the mesial temporal lobe, with 50% growth compared to a previous study. With these results, the patient was referred to our institution where a review of the pathology plaques and a cerebral MRI with spectroscopy was requested. The pathology reports fusocellular mesenchymal neoplasia with smooth muscle differentiation by immunohistochemistry, compatible with low-grade leiomyosarcoma.

**Figure 1:**
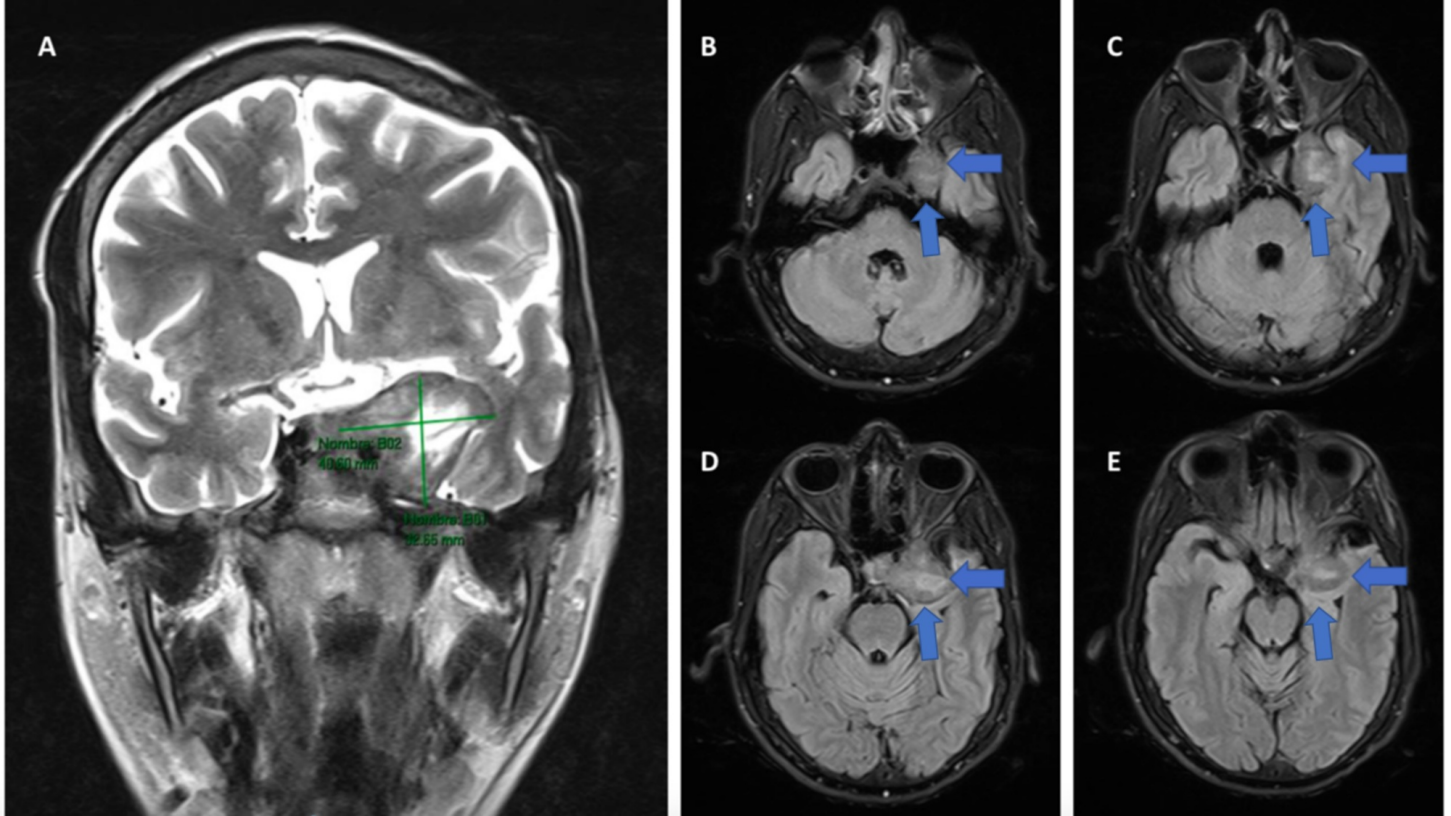
Cerebral MRI (A) Coronal section showing the dimensions of the tumor. (B) Axial section of the lower part of the tumor. (C) Axial section where the relationship of the mass with the skull base structures begins to be shown. (D) Axial section showing how the mass infiltrates the cavernous sinus. (E) Axial section of the upper part of the tumor.

**Figure 2:**
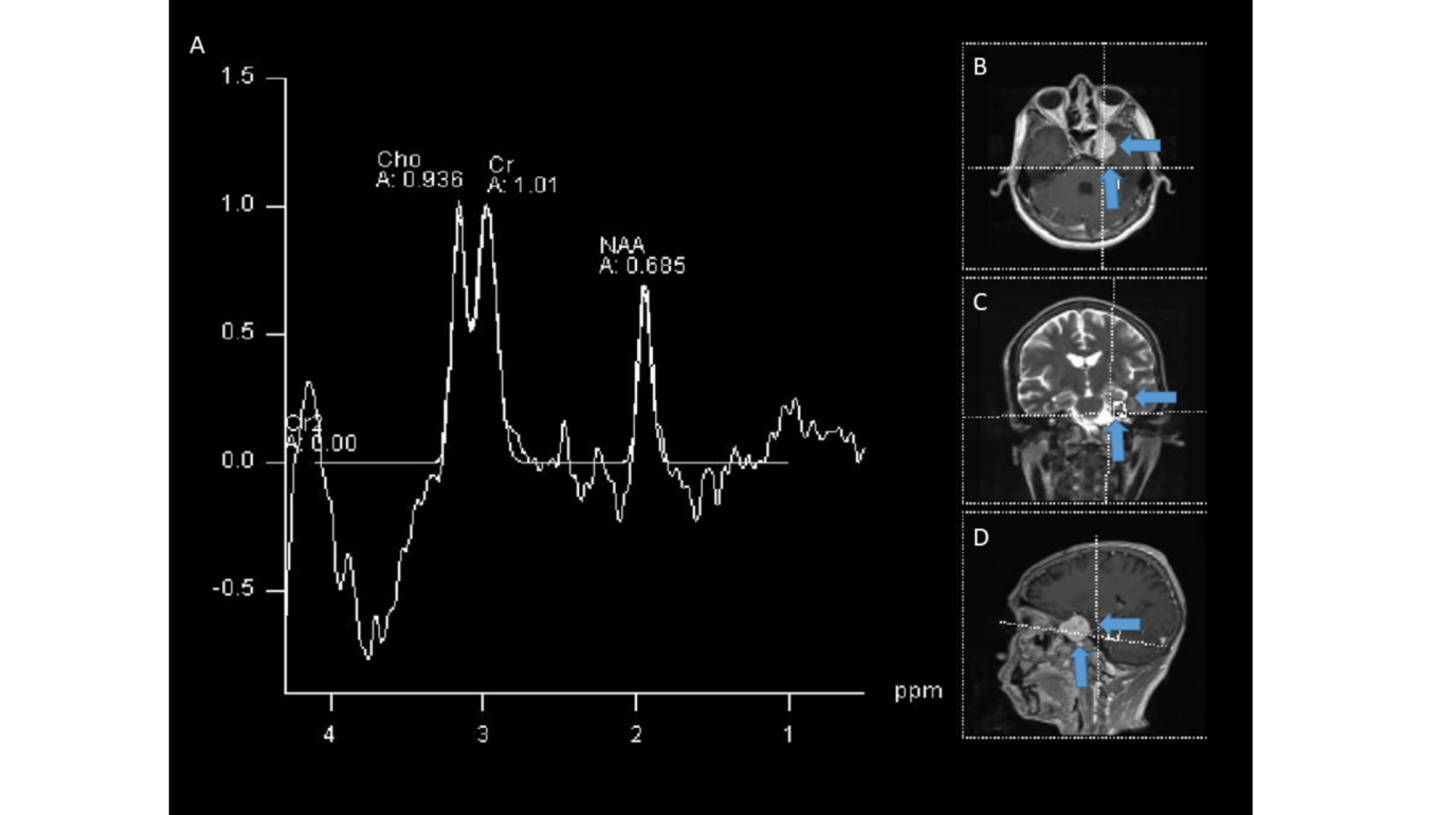
Spectroscopy (A) The study showed decreased levels of neuronal metabolic components choline (Ch), creatine (Cr) and N-acetylaspartate (NAA) with a significant increase in the lactate and lipid levels. (B) Axial location of the tumor. (C) Coronal location of the tumor. (D) Sagittal location of the tumor.

Immunohistochemistry reported Vimentin (+), SMA (+), H-Caldesmon (+) focal, KI67:15%, CKAE1AE3 (-), CD34 (-), STAT-6 (-), Chromogranin (-), Synaptophysin (-), EMA (-), S100 (-), PGFA (-), Progestogen (-), Desmin (-), PGP9.5 (-), CD99 (-), CD31 (-), HHV8 (-), ACL (-). In addition to this, a fluorescence in situ hybridization (FISH) test was performed, which was Epstein-Barr virus-encoded small ribonucleic acid (EBER) positive confirming coinfection by Epstein-Barr virus (EBV). In extra axial location with epicenter in the cavernous sinus and with extension to the medial temporal fossa on the left side, the MRI (Figure 1) identified a solid mass of well-defined and irregular contours, with low signal intensity to T2-enhanced sequences, without significant restriction in the diffusion sequences, with intense enhancement after intravenous contrast administration and with central focal areas of necrosis and/or malacia due to the history of partial resection, and surrounding the entire cavernous portion of the internal carotid artery without secondary occlusion. This lesion has diameters greater than 32 x 35 x 46 mm. In the spectroscopy sequences (Figure 2), decreased levels of neuronal metabolic components choline (Cho), creatine (Cr) and N-acetylaspartate (NAA) were identified with a significant increase in the values of lactate and lipid levels attributable to anaerobic activity.

**Figure 3:**
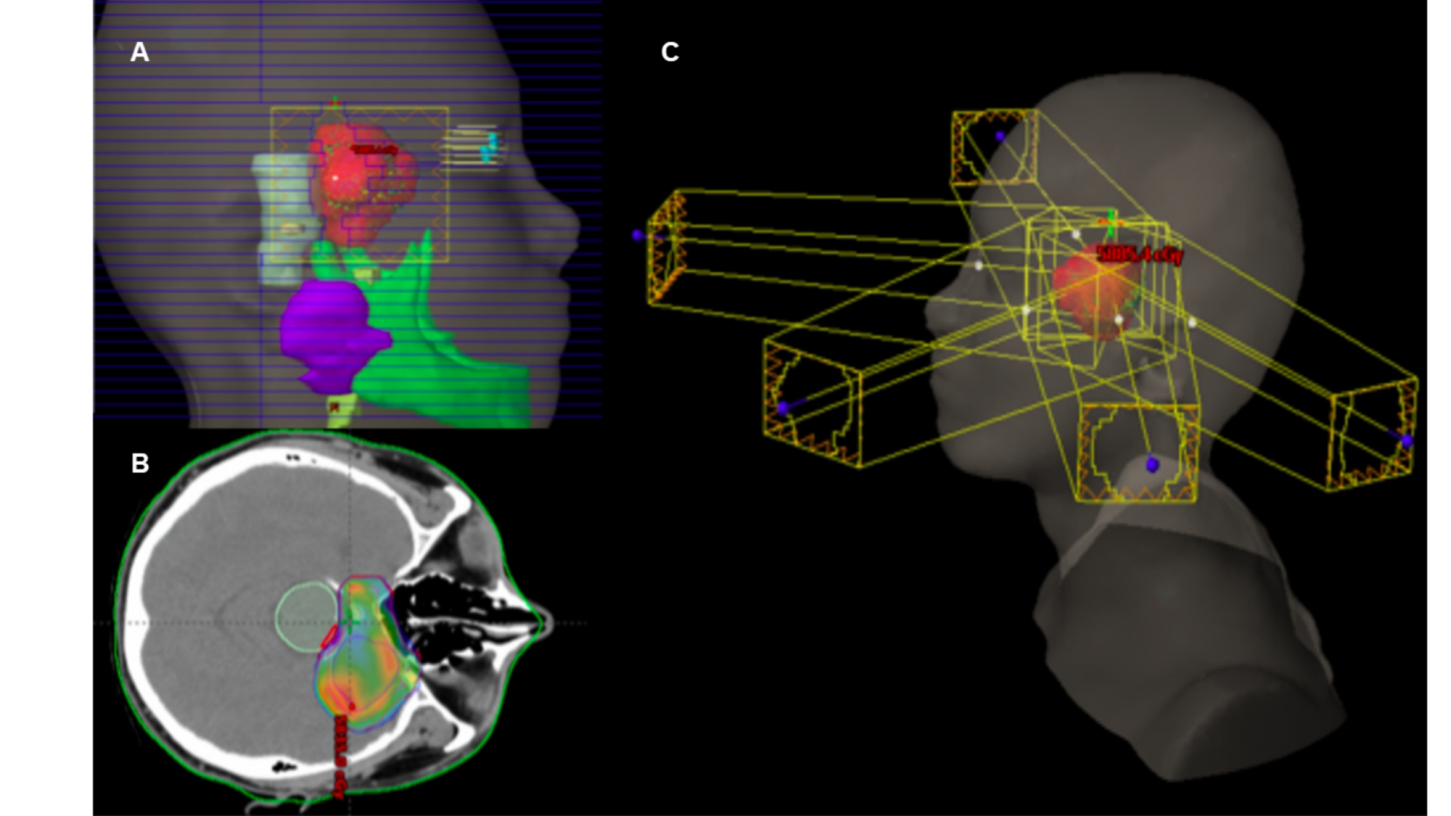
Treatment of the patient with intensity-modulated radiation therapy (IMRT) (A) Three-dimensional reconstruction of the lateral view of the delimited volumes for treatment. (B) Axial section showing the distribution of the radiation dose on the tumor. (C) Radiotherapy field arrangement.

The patient was assessed by the board of neurosurgery, who considered that the lesion is unresectable due to its location and the risk of motor sequelae after treatment. Clinical oncology considered that given the low tumor grade, the patient should be initially treated with radiotherapy. The patient was presented to the radiotherapy board, which defined treatment with intensity-modulated radiation therapy (IMRT) technique external radiotherapy (Figure 3), total dose of 54 Gy, in fractionation of 1.8 Gy. The patient completed treatment referring headache improvement without evident secondary toxicity. Six months after the treatment, the patient continued asymptomatic with stable disease.

## Discussion

Primary intracranial leiomyosarcomas are extremely rare tumors with less than 50 cases reported [3]. Primary intracranial smooth muscle tumors can arise from the leptomeningeal coatings, with the pluripotent mesenchymal stem cells in the dura-mater being the probable origin cells [5]. Other reports suggest that epithelium cerebral blood vessels could be the site of origin of these sarcomas [5-7]. Its age of onset is quite wide since there are reports in patients with ages ranging from 4 to 75 years [3,6-8]. There seems to be a predilection for male patients with a ratio of 2.3:1.6 [8]. The average duration of symptoms before diagnosis is approximately four months; these symptoms are closely related to the location of the tumor [9]. According to Zhang et al. report, out of 37 patients, 18 (48.6%) had the dura as the tumor’s site of origin, 10 (27.0%) had it in the convex set, five (13.5%) at the base of the skull and three (8.1%) in the tentorium cerebelli [8]. In the available reports we only found two patients who had, as in our case, the cavernous sinus as a site of origin [10,11]. This is particularly important because it limits the possibilities of treatment, since of these three patients, one was treated exclusively with 15 Gy radiosurgery in five fractions [11], and the other reported patient, like ours, was taken to surgery but a complete resection was not achieved [10].

An important point to note is that about 43.2% of intracranial leiomyosarcomas are related to immunosuppression; of the reported cases, only 13 are associated with HIV infection and of these, eight were related to EBV [8]. This association of leiomyosarcomas and HIV has been increasing in the last 10 years and it has been suggested that EBV can infect smooth muscle, at least in patients with acquired immunodeficiency syndrome (AIDS), and contribute to the pathogenesis of leiomyosarcomas [6].

The prognosis of these patients is generally bad, being the patient with the longest follow-up time, and one who died at 44 months [12]. This may probably be associated with the fact that, since it is a rare tumor, there is no standard treatment and localization limits local therapies [6,8]. Even so, it has been reported that about 90% of patients are taken to surgery as initial therapy [4,8,12], and of these patients, approximately 54.1% will be taken to adjuvant radiotherapy and 18.9% will be treated with adjuvant chemotherapy. The role of the latter is not clear since many of the agents used in sarcoma do not cross the blood-brain barrier or have a moderate activity [6]. Of the reported cases, only two were treated exclusively with radiosurgery [11,12]. In our case, although it was initially treated with a partial resection, it was not until one year later, when documenting the progression of the disease, that the patient was taken to treat the progression exclusively with radiotherapy up to a dose of 54 Gy not to exceed the tolerance dose of the organs at risk.

## Conclusions

According to the evidence reviewed, primary leiomyosarcomas of the central nervous system are extremely rare tumors that can affect pediatric patients to the elderly. They have become important given their increase in recent years, especially in relation to HIV infection. Although it is a fairly rare condition that does not have a defined standard treatment, in most of the available reports these patients are initially treated with surgery and some are subsequently taken to an adjuvant treatment. Radiation therapy is an adjuvant option often used in studies, it is also important in patients with unresectable disease, relapse or progression as is the case with our patient. Unfortunately, the prognosis of these patients is bad and we currently do not have enough information that allows us to define the best management strategy, although the available reports suggest that the treatment should be performed by a multimodal group.
